# Transcatheter Mitral Valve Replacement with Dedicated Devices

**DOI:** 10.14797/mdcvj.1231

**Published:** 2023-05-16

**Authors:** Joe Aoun, Michael J. Reardon, Sachin S Goel

**Affiliations:** 1 Houston Methodist DeBakey Heart & Vascular Center, Houston Methodist Hospital, Houston, Texas, US

**Keywords:** mitral regurgitation, transcatheter mitral valve replacement, Tendyne, Intrepid, Sapien valve

## Abstract

Mitral regurgitation is the most common form of valvular heart disease. The anatomy and pathophysiology of mitral valve regurgitation are very complex, and dedicated devices are required for transcatheter mitral valve replacement in patients with a high or prohibitive surgical risk. In the United States, all transcatheter mitral valve replacement devices are still being studied and are not yet approved for commercial use. Early feasibility studies have demonstrated good technical success and short-term outcomes, but larger samples and longer-term outcomes still need to be assessed. Furthermore, significant advances in device technology, delivery systems, and implantation techniques are essential to avoid left ventricular outflow tract obstruction, and valvular and paravalvular regurgitation as well as ensuring good anchoring of the prosthesis.

## Introduction

Mitral regurgitation (MR) is the most common form of valvular heart disease. In the current era, the management of MR is determined by several factors, including etiology, severity, and surgical risk of the patient. Untreated severe MR can result in high mortality rates.^1^ However, only 84% of primary MR patients and 36% of secondary MR patients undergo surgical interventions, leaving an unmet need for treatment options for patients who are high-risk for surgery. This has led to the development of transcatheter options including transcatheter-edge-to-edge repair (TEER) and transcatheter mitral valve replacement (TMVR).^1,2^

Early advancements in transcatheter aortic valve replacement (TAVR) contributed to the use of aortic transcatheter heart valves in the mitral position. Successful implantations were noted in patients with failed bioprosthetic valves (valve-in-valve), annuloplasty rings (valve-in-ring), and mitral annular calcification (MAC), but not in native mitral valves without MAC. For this reason, significant efforts were undertaken for designing new devices that implanted into the native mitral annulus. Dedicated TMVR devices are needed to provide anchoring, eliminate MR, and reduce the risk of paravalvular regurgitation and left ventricular outflow tract (LVOT) obstruction.

## Challenges in Tmvr

TMVR devices face several challenges. The mitral annulus is a large D-shaped structure defined anteriorly by the aortomitral curtain, making device sizing a challenging process. The annulus size is larger than the aortic, thus larger devices are needed that have higher-profile delivery systems. This translates into larger iatrogenic atrial septal defects for transseptal delivery systems with possible right to left shunting and resulting hypoxia. The delivery catheter needs flexibility to reach the mitral implant position. In the absence of mitral annular calcification, an anchoring mechanism is required, especially since the anterior aortomitral curtain is a dynamic and flexible structure. Finally, the proximity of the device to the LVOT and the presence of the anterior mitral valve leaflet during TMVR can induce LVOT obstruction, which appears to be the Achilles heel of TMVR.

Clinical trial design for TMVR poses several challenges. The obvious major benefit of TMVR is MR elimination, which is in contrast to TEER; however, this needs to be evaluated against its risks. Randomized controlled trials have provided the highest quality data for safety and effectiveness of novel transcatheter devices over the last decade. For TMVR, standard of care treatment that can serve as appropriate control group in a randomized controlled trial is evolving. For TMVR in patients with primary MR, randomization must be considered against surgical mitral valve repair or TEER for patients at high surgical risk. For secondary MR, TMVR must be randomized against guideline-directed medical therapy or TEER, depending on the clinical situation and anatomy, given that TEER now has a class 2A guideline recommendation in this setting.

Randomization of patients with secondary MR to surgery is difficult given lack of guideline recommendations and favorable surgical data. Single arm TMVR evaluation may be appropriate in the context of overall randomized controlled trial design when randomization to surgery or TEER is not feasible or possible due to clinical or anatomic reasons. An important consideration of TMVR trials is the appropriate evaluation of surgical candidacy, contemporary guideline directed medical therapy (especially in secondary MR), and evaluation of TEER suitability, which is supported by guidelines for high-risk primary MR and medically optimized secondary MR patients. With regard to trial end points, the primary end point for TMVR trials should include those related to heart failure, such as mortality and/or heart failure hospitalizations. Quality of life end points (Kansas City Cardiomyopathy Questionnaire score) may be considered as a component of a composite primary end point as suggested in the Mitral Valve Academic Research Consortium document. Randomized trials with TEER as the control group can be designed to demonstrate superiority or noninferiority; however, those against guideline-directed medical therapy must be designed to demonstrate superiority. Secondary end points should include those pertaining to safety, measures of success, left ventricular remodeling, and functional capacity.

**Figure 1:**
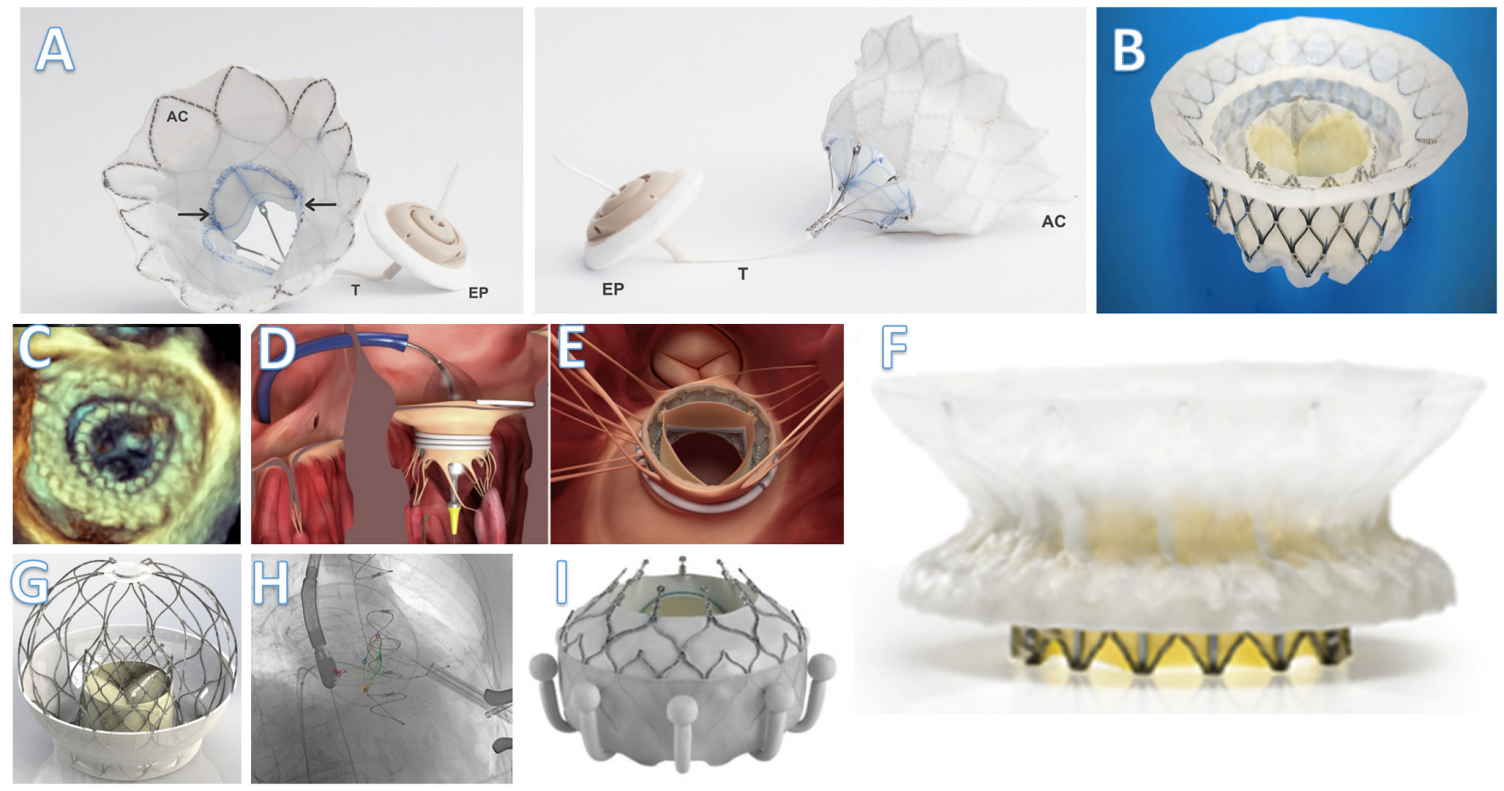
Transapical and transseptal transcatheter mitral valve replacement (TMVR) systems. (A) Tendyne valve, courtesy of Abbott; (B) Intrepid valve, courtesy of Medtronic; (C) three-dimensional transesophageal echocardiogram showing the Intrepid valve after implantation; (D) Sapien M3 valve, transseptal placement of the docking system (both D and E permission obtained from Elsevier; (E) surgeon’s view of the Sapien M3 after placement; (F) Cephea valve, courtesy of Abbott; (G) AltaValve, courtesy of 4CMedical; (H) fluoroscopic appearance of the AltaValve during TMVR; and (I) Evoque valve, courtesy of Edwards Lifesciences. AC: outer frame with a cuff; EP: epicardial pad; T: Tether. From Aoun J et al. Transcatheter mitral valve replacement: an update. Curr Opin Cardiol. 2021 Jul;36(4):384-89. Copyright ©2021 Wolters Kluwer Health, Inc. All rights reserved.

Currently, several TMVR systems (transapical or transseptal) are being evaluated in multiple feasibility studies and pivotal trials. The delivery mechanism, deployment, and anchoring mechanisms vary significantly between devices. Devices can be annular, supra or subannular, with or without a docking system (mimicking a “valve-in-ring” concept), and with or without (annular or ventricular) anchors. We hereby discuss TMVR devices that are currently being studied (Figure 1).

## Tendyne

The Tendyne TMVR system (Abbott Vascular) is an intra-annular, self-expanding nitinol bioprosthesis with a trileaflet porcine pericardial valve. The system consists of an outer frame that serves as a supra-annular cuff to prevent paravalvular MR. It is a retrievable and repositionable device delivered transapically via a 36F sheath and anchored to the left ventricular apex by an epicardial pad tether. It is possible to tighten the tether for a few days up to weeks after implantation to decrease any significant development of paravalvular leak.^3,4^

The Tendyne TMVR system is the most studied device thus far, with the largest published cohort of 100 patients followed up to 2 years.^5,6,7^ The device was founded in 2010, acquired by Abbott in 2015, and approved for CE mark in Europe since January 2020 after the publication of the largest early feasibility, multinational, nonrandomized study. This study included 100 patients with ≥ 3+ MR at high to prohibitive surgical risk who underwent implantation of the Tendyne valve.^7,8^ According to the Mitral Valve Academic Research Consortium definitions, technical success was achieved in 96% of the patients, along with 94% and 72.4% 30-day and 1-year survival rates, respectively. The majority of patients had NYHA I/II at 1 year (88.5%).

The SUMMIT trial (Clinical Trial to Evaluate the Safety and Effectiveness of Using the Tendyne Mitral Valve System for the Treatment of Symptomatic Mitral Regurgitation) is an ongoing large randomized study to compare Tendyne TMVR with TEER using MitraClip in patients with ≥ 3+ MR.^9^ Patients with severe MAC (≥ 3+ MR or severe MS or moderate MR with at least moderate MS) will be enrolled in a nonrandomized arm. The primary outcome includes 1-year survival free of heart failure hospitalization and the composite of all-cause mortality, cardiovascular-related hospitalizations, stroke, or mitral valve reintervention or reoperation.^9^

## Intrepid

The Intrepid TMVR system was developed by Foundry Newco XII, Inc, and was acquired by Medtronic in 2015. It consists of a 27-mm trileaflet bovine pericardial valve surrounded by an internal frame as well as an outer fixation frame with a flexible atrial segment and a stiffer ventricular segment in sizes of 42 and 48 mm, with a 54-mm valve in development.^10^ The system is delivered via a 35F straight or a 37F curved sheath and can be implanted via a transapical or transseptal approach.

The first early feasibility study, the Intrepid Global Pilot study, was published in 2018 and included 50 patients at high surgical risk who had compatible anatomy for the device.^11^ Important exclusion criteria included LVEF < 20% and severe pulmonary hypertension with systolic pulmonary pressure > 70 mm Hg. All patients underwent a transapical approach with the Intrepid valve TMVR and had Society of Thoracic Surgeons (STS) and EuroSCORE scores of 6.4% +/- 5.5% and 7.9% +/- 6.2%, respectively. One patient did not receive the valve due to left ventricle apical bleeding. The majority of patients (72%) had secondary MR, while 16% had primary MR and 45% had at least moderate TR. Technical success was achieved in 98% of patients, and there were no device malfunctions or failures or conversions to open cardiac surgery. The survival rate at 30 days was 86%, with the majority having less than mild MR.^11^

In addition, a prospective, multicenter, nonrandomized study enrolled 15 patients from six different sites and demonstrated a procedural success rate of 93% (14 out of 15 patients) and a 30-day survival rate of 100% with a transfemoral, transseptal insertion approach.^12^

The APOLLO trial (Transcatheter mitral valve replacement with the Medtronic Intrepid™ TMVR system in patients with severe symptomatic mitral regurgitation) is an ongoing large nonrandomized open-label trial.^13^ The study evaluates the outcomes of TMVR in native valves and the outcomes of TMVR in patients with MAC in a separate arm. The primary outcome includes a composite of all-cause mortality, heart failure hospitalization at 30 days, and KCCQ improvement.^13^

## Altavalve

The AltaValve (4C Medical Technologies) is a supra-annular valve delivered transapically via a 32F sheath. A transfemoral delivery system has been developed and now is being studied. The system is comprised of a 27-mm trileaflet bovine pericardial valve encased in a spherical nitinol frame and a fabric skirt on the bottom at the level of the annular ring (available ring sizes: 40, 46 and 54 mm). Due to its unique supra-annular position, the risk of LVOT obstruction and embolization may be lower than other devices.

The AltaValve is being evaluated in an early feasibility study. Limited results were presented in the American Association for Thoracic Surgery AATS 2022 Mitral Conclave Workshop and demonstrated 100% technical success rate in 10 patients. Several successful cases of AltaValve implant have been described in the literature.^14,15^

## Tiara

The Tiara valve (Neovasc Inc.) is a self-expanding, nitinol-based frame with bovine leaflets requiring a 32F or 36F delivery sheath. The available valve sizes include 35 mm and 40 mm. The valve stability relies on ventricular anchoring tabs.

The Tiara-I study enrolled 27 patients with severe MR who underwent implantation of the Tiara valve system and demonstrated an 89% survival rate at 30 days.^16^ Neovasc has filed for CE mark in 2020 but still remains under clinical investigation.

## SAPIEN M3

The SAPIEN M3 TMVR system (Edwards Lifesciences) is a balloon expandable bovine pericardial valve implanted in a nitinol docking system via a transseptal approach.^17^ The docking system encircles the mitral chordae to provide support for valve implantation.

The results of the first-in-human study demonstrated successful implantation of the Sapien M3 system in 9 out of 10 patients.^15^ There was significant MR reduction (less than mild residual) in all patients without a significant increase in mitral valve gradient (mean gradient of 2.3 mm Hg).

The results of the early feasibility study were presented in the Transcatheter Cardiovascular Therapeutics 2019 meeting.^18^ Thirty-five patients with severe MR and high surgical risk underwent TMVR using the Sapient M3 system, with successful implant seen in 89% (31/35 patients). Paravalvular regurgitation occurred in 2.8% (1/35 patients) requiring intraprocedural closure.^18^ The 30-day survival rate was 97.1%, and 93.8% of the patients had ≤ 1+ MR with a mean mitral gradient of 5.36 mm Hg.^18^

## EVOQUE Mitral System

The EVOQUE TMVR system (Edwards Lifesciences) consists of a bovine pericardial valve in a nitinol frame with ventricular anchors and an intra-annular sealing skirt to decrease the risk of paravalvular leak.^17^ It is implanted transeptally using a 28F sheath. Available sizes include 44 mm and 48 mm.

The first-in-human experience using the EVOQUE TMVR system studied 14 patients with severe MR with at least high surgical risk.^19^ The technical success of the procedure was around 93%. The majority of PVL cases were mild except in one patient requiring percutaneous closure and another requiring conversion to surgical mitral valve replacement. Only one patient developed LVOT obstruction. Survival rate at 30 days was 93%. There was significant symptomatic improvement seen in NYHA classification and KCCQ-12 score.^19^

The safety and performance of the valve is currently being evaluated in the MISCEND study—Edwards EVOQUE Eos Mitral Valve Replacement: Investigation of Safety and Performance After Mitral Valve Replacement With Transcatheter Device.^20^

## Cephea

The Cephea TMVR system (Abbott Vascular) consists of a self-expanding bovine pericardial valve (sizes 32, 36 or 40 mm) encased in a nitinol double disc frame and delivered via a transseptal approach.^21^ The anchoring mechanism is based on axial compression force.

The first valve was implanted in 2019.^22^ Subsequently, three patients with severe degenerative MR and prohibitive surgical risk underwent successful implantation of the Cephea device. There was a significant MR improvement (≤ mild MR) with significant increase in transmitral gradient (mean gradient of 3 mm Hg) and no LVOT obstruction. To our knowledge, 10 patients have received the Cephea TMVR device. An early feasibility study is currently ongoing in the US.

## Highlife

The HighLife TMVR system (HighLife SAS) is a self-expanding nitinol subannular valve with bovine pericardial leaflets implanted in a ring. It is available in a 31-mm valve size. Transseptal delivery is achieved using a 39F sheath. The subannular ring is implanted via a transaortic retrograde approach.

The first interim data was presented during the Transcatheter Cardiovascular Therapeutics 2021 meeting and demonstrated a 90% technical success rate (27/30 patients).^23^ Surgical conversion was described in one patient. At 30 days, the mortality rate was 10%, and 75% had less than mild MR.^23^ Currently, an ongoing early feasibility trial is underway.^24^

## Innovalve

The Innovalve TMVR system is a novel transseptal TMVR system consisting of an atrial flange and a cylinder with six arms designed for grasping and wrapping the chordae followed by positioning, anchoring, and delivery of the valve.^25^ The TWIST-EFS study aims to enroll 15 participants with significant MR and high operative risk to undergo TMVR with the Innovalve system.^26^ This system has the potential of reducing LVOT risk by holding the anterior leaflet away from the LVOT by way of the rotation and chordal wrapping mechanism.

## Conclusion

Mitral regurgitation is a common valvular disease with complex anatomy and pathophysiology. Despite significant advances in transcatheter aortic devices, dedicated devices in the mitral position are essential and currently being studied. Success in the TMVR field requires excellence in cardiac imaging and patient selection in addition to future advances in valve anchoring, decreases in delivery sheath profiles, and formation of prediction models to decrease LVOT obstruction.

## Key Points

- Transcatheter mitral valve replacement (TMVR) in high-risk surgical patients with severe native mitral valve regurgitation is feasible using dedicated devices.
- To date, the Tendyne TMVR system is the only system with CE Mark.
- In the United States, all TMVR systems are being studied and are not ready for commercial use outside of clinical trials.
- Significant advances in device technology, delivery systems, and implantation techniques are essential to avoid vascular complications, left ventricular outflow tract obstruction, and achieve good anchoring to reduce paravalvular regurgitation.
